# Development and validation of a novel coronary artery disease risk prediction model

**DOI:** 10.1186/s12967-024-05789-1

**Published:** 2025-01-10

**Authors:** Zu-Fei Wu, Si-Xiao Tao, Wen-Tao Su, Shi Chen, Bai-Da Xu, Gang-Jun Zong, Gang-Yong Wu

**Affiliations:** 1 Department of Cardiology, Xuancheng People’s Hospital, Xuanchen, Anhui 242000 People’s Republic of China; 2 Department of Cardiology, The 904th Hospital of the PLA Joint Logistics Support Force, Wuxi, Jiangsu 214044 People’s Republic of China; 3 Department of Cardiology, Wuxi Clinical College of Anhui Medical University, Wuxi, Jiangsu 214044 People’s Republic of China; 4 Department of Cardiology, Wuxi No.5 People’s Hospital, Wuxi, Jiangsu 214044 People’s Republic of China

**Keywords:** Coronary artery disease, Risk assessment, Inflammation

## Abstract

**Objective:**

This study aims to develop a novel risk assessment tool for coronary artery disease (CAD) based on data of patients with chest pain in outpatient and emergency department, thereby facilitating the effective identification and management of high-risk patients.

**Methods:**

A retrospective analysis was conducted on patients hospitalized for chest pain. Patients were divided into a control group and a CAD group based on angiographic results. Logistic regression was used to identify factors associated with CAD, and R-Studio was utilized to construct the CAD risk prediction model.

**Results:**

Multivariate logistic regression analysis indicated that age, gender, diabetes, ECG (electrocardiogram) ST-T changes, neutrophils (NE), coronary artery calcification (CAC), and typical chest pain were independent factors associated with CAD. Based on the results of multifactorial logistic analysis, the CAD risk prediction model built with R-Studio had a highest C-index of 0.909, and a validation cohort C-index of 0.897, demonstrating excellent predictive ability. Decision Curve Analysis showed that the model significantly outperformed others in terms of clinical net benefit.

**Conclusion:**

The present study successfully developed a CAD risk assessment model based on Chinese population. This novel model could be used to assess CAD risk in patients with chest pain, optimize clinical decision making, and improve patient outcomes, regardless of whether it is applied in large hospitals or resource-limited Community Healthcare Center.

**Supplementary Information:**

The online version contains supplementary material available at 10.1186/s12967-024-05789-1.

## Introduction

Cardiovascular disease is one of the largest global health burdens, claiming nearly 17 million lives each year [1]. Coronary artery disease (CAD) is the primary cause of cardiovascular diseases, posing a severe threat to human life and placing a significant economic burden on society [2]. Matthew J Budoff et al. found that among patients undergoing invasive coronary angiography with clinical indications, 41% had obstructive coronary lesions [3]. Each year, millions of patients worldwide visit hospitals at various levels due to chest pain discomfort, and risk assessment for these patients has always been a challenging clinical issue [4].

In assessing the risk of CAD, it is critical to accurately distinguish between low- and high-risk patients. Low-risk patients typically have better cardiovascular health and fewer common risk factors, such as a lower prevalence of smoking, hypertension, and diabetes. In contrast, high-risk patients may present multiple risk factors simultaneously, significantly increasing the likelihood of cardiovascular events, which necessitates more aggressive intervention and monitoring [5].

Cardiologists can rely on their clinical experience and basic clinical tools (history, physical examination, and electrocardiogram) to meet this challenge and identify high-risk chest pain patients early. However, for non-cardiologists, emergency physicians, and cardiologists with limited clinical experience, identifying high-risk chest pain patients remains a significant challenge. Although most of these patients do not have life-threatening diseases, clinicians must distinguish between high-risk patients who need urgent treatment and low-risk patients who do not require hospitalization. Incorrect risk assessment of coronary heart disease can lead to high-risk patients developing acute coronary syndrome or even death after leaving the hospital, increasing the liability risk for attending physicians. Hospitalizing low-risk patients without indication is neither cost-effective nor necessary, causing an unnecessary waste of medical resources.

To address this challenge, an increasing number of diagnostic strategies and methods have been applied in clinical practice, such as chest pain units (CPU), cardiac biomarkers, accelerated diagnostic protocols, and non-invasive imaging of the myocardium and coronary arteries [6]. Although these methods have made some progress in improving the diagnostic accuracy and management of chest pain patients, each has its limitations.

Chest pain units and accelerated diagnostic protocols mainly focus on identifying high-risk patients, failing to comprehensively cover all chest pain patients [7]. Cardiac biomarkers have a specific time window and may produce false positives or false negatives under certain circumstances, affecting diagnostic accuracy [8–10]. Although non-invasive imaging techniques provide detailed anatomical and functional information, their high cost and complexity limit their widespread use in primary healthcare institutions [11, 12].

Each of these methods has certain limitations and cannot cover all chest pain patients. Risk scoring is a simple, convenient, and widely applicable method. These risk scoring tools are based on multiple clinical and laboratory parameters and calculate the patient's risk score to help clinicians make accurate judgments in a short time. Currently, pre-test probability (PTP) models related to coronary heart disease include the Diamond and Forrester model [13], PTP model [14], and CACS-CL tool [15]. These risk tools were developed based on Western populations and have certain limitations in their application, making them unsuitable for Asian populations. Therefore, there is an urgent need for a clinically applicable coronary heart disease risk assessment tool for Asian populations, especially the Chinese population, to meet this clinical need. Based on routine examinations and specific symptoms of chest pain patients at outpatient and emergency visits, we can quickly and accurately assess the patient's risk of coronary heart disease. This helps clinicians better identify high-risk patients, optimize treatment decisions, avoid unnecessary hospitalization and waste of medical resources, and ultimately improve patient outcomes.

This study aims to develop a new risk assessment tool based on routine examinations and specific symptoms of chest pain patients at outpatient and emergency visits, to more effectively identify and manage high-risk chest pain patients, optimize the use of medical resources, reduce unnecessary hospitalizations, and ultimately improve patient outcomes.

### General information

Using a retrospective analysis method, a total of 2756 patients who presented with chest pain at the outpatient or emergency department of Xuancheng People's Hospital from January 2020 to December 2022 were identified from the Hospital Information System. Relevant laboratory tests, electrocardiograms, and imaging examination results for these patients at the time of their outpatient and emergency visits were collected. This study was reviewed and approved by the ethics committee. Inclusion criteria: (1) patients with suspected obstructive coronary heart disease admitted for elective coronary angiography in outpatient or emergency settings; (2) patients who were conscious, able to communicate naturally, and free of severe neurological and psychiatric diseases. All subjects received standardized dual antiplatelet therapy with aspirin and ticagrelor/clopidogrel after admission. Exclusion criteria: (1) patients with a history of myocardial infarction, coronary stent implantation, or coronary artery bypass grafting; (2) patients with acute cerebral infarction within the past six months; (3) patients diagnosed with pulmonary embolism, aortic dissection, acute or chronic nephritis, or other systemic diseases; (4) patients with hematologic disorders, malignant tumors, or autoimmune diseases; (5) patients with acute or chronic infectious diseases. Finally, 2,100 patients were included. Using R language, all patients were randomly divided into the experimental cohort and validation cohort (7:3), with 1,470 patients in the experimental cohort and 630 patients in the validation cohort. Parameters with less than 5% missing data were imputed using multiple imputation, and those with more than 5% missing data were deleted. All patients signed informed consent upon admission, agreeing to the use of their medical data for clinical research purposes.

## Methods

All relevant clinical data for outpatient and emergency patients were collected, including age, sex, and symptoms of chest pain. Upon presentation to the outpatient or emergency department, patients' systolic and diastolic blood pressures were recorded. Hypertension, diabetes, and smoking status were documented as present or absent. Within 20 min of the outpatient or emergency visit, venous blood was drawn from all patients for routine blood tests. Serum creatinine and blood urea nitrogen levels were measured using an automatic biochemical analyzer (Beckman, USA). Blood routine examination results were checked using Sysmex(XA-2800). All patients underwent a 12-lead electrocardiogram (ECG) within 10 min (DMS, DMS300BTT02) and a chest CT scan within 30 min (UNITED IMAGING, uCT530). Reports were reviewed and archived by at least two experienced physicians.

### Clinical definitions

Type of chest pain was classified as being typical, atypical, or non-specific. Typical chest pain was defined as having (i) substernal chest pain or discomfort, that is (ii) provoked by exertion or emotional stress and (iii) relieved by rest and/or nitroglycerine. Atypical chest pain was defined as having two of the before-mentioned criteria. If one or none of the criteria was present, the patient was classified as having non-specific chest pain [16].

The presence of obstructive CAD was defined as one or more vessels with ≥ 50% lumen diameter reduction on CAG(Coronary Angiography). As we used existing databases, CAG was performed at each institution according to local protocols; both visual assessment and quantitative assessment were allowed for interpretation of the CAG. Indicator variables for hospital were used to allow adjustment for hospital [17].

Two professional radiologists provided a simple, overall visual assessment of the chest CT images to check for the presence of coronary artery calcification in the entire coronary arterial circulation [18].

### Patient grouping

Using R-Studio, all patients were randomly divided into the experimental cohort and validation cohort (7:3). The experimental cohort included 1,470 patients, divided into a control group of 589 patients and an obstructive CAD group of 881 patients based on coronary angiography results. The validation cohort included 630 patients, divided into a control group of 243 patients and a CAD group of 387 patients.

### Statistical methods

Analysis was performed using SPSS 26.0 statistical software. Quantitative data conforming to a normal distribution were expressed as mean ± standard deviation (Mean ± SD), while non-normally distributed data were expressed as median (interquartile range) [M (Q25, Q75)]. The Mann–Whitney U test was used for comparisons between non-normally distributed sample groups, and independent sample t-tests or one-way ANOVA were used for normally distributed sample groups. Qualitative data were expressed as counts and percentages, and comparisons between groups were performed using the chi-square test. Univariate and multivariate logistic regression analyses were conducted to identify independent factors associated with CAD. Based on the results of multivariate logistic regression analysis, a nomogram was constructed using the rms package (version 6.8–0) in R-studio statistical software (version 4.1.2) and the C-index was calculated. Each patient's score was computed, and a scoring table was constructed. The rmad package (version 1.6) was used for decision curve analysis (DCA) to evaluate the clinical application value and net benefit of the CAD risk score.

## Results

The final experimental cohort included 1,470 patients, with 589 in the control group and 881 in the obstructive CAD group. The validation cohort included 630 patients, with 243 in the control group and 387 in the CAD group. Specific demographic and clinical characteristics are shown in Table 1.

**Table 1:** Demographics and clinical characteristics in the derivation and external validation cohorts

Characteristic	Derivation (N=1470)	Validation (N=630)	*Z/T/X*	*P*
Age, (years)	59 (53,70)	59 (53,70)	−0.162	0.872
Weight, (Kg)	64 (57,72)	64 (57,72)	−0.023	0.981
Heart rate, (Bpm)	76.5 (68,87)	76 (69,85)	−1.287	0.198
SBP, (mmHg)	138 (126,152)	138 (125,151)	−0.269	0.788
DBP, (mmHg)	84 (77,93)	84 (76,92)	−0.776	0.438
AST, (U/L)	20.6 (17.0,26.1)	20.6 (16.575,27.025)	−0.289	0.773
BUN, (mmol/L)	5.38 (4.46,6.47)	5.40 (4.51,6.4125)	−0.804	0.421
Ca, (mmol/L)	2.32 (2.25,2.40)	2.32 (2.24,2.3925)	−0.808	0.419
Scr, (umol/L)	67.4 (56.5,78.6)	68.8(57.825,81.0)	−2.031	0.042
ABG, (mmol/L)	5.3 (4.9175,5.94)	5.3 (4.93,5.8525)	−0.536	0.592
UA, (umol/L)	333.0 (277.0,392.25)	339.0 (281.0,400.0)	−1.42	0.156
FIB, (mmol/L)	3.17 (2.78,3.7)	3.17 (2.78,3.6525)	−0.174	0.862
HGB, (g/L)	134 (124,145)	135 (124,147)	−1.213	0.225
LY, (×10^9^/L)	1.54 (1.22,1.95)	1.56 (1.2275,1.94)	−0.448	0.654
MONO, (×10^9^/L)	0.41 (0.32,0.51)	0.42 (0.3275,0.52)	−1.516	0.129
NE, (×10^9^/L)	3.77 (2.97,4.7925)	3.83 (3.0075,4.8725)	−1.229	0.219
PLT, (×10^9^/L)	190.5 (155,232)	188 (153.75,233)	−0.234	0.815
RBC, (×10^9^/L)	4.48(4.13,4.87)	4.51 (4.12,4.93)	−1.075	0.282
WBC, (×10^9^/L)	6(4.95,7.2125)	6.025 (5.12,7.335)	−1.453	0.146
Male, n (%)	808 (54.97)	383 (60.79)	6.101	0.014
Hypertension, n (%)	831 (56.53)	362 (57.46)	0.155	0.693
Diabetes, n (%)	247 (16.80)	110 (17.46)	0.135	0.713
Smoking, n (%)	283 (19.25)	140 (22.22)	2.419	0.12
Cerebral Infarction, n(%)	174 (11.83)	76 (12.06)	0.022	0.883
Hyperlipidemia, n(%)	611 (41.56)	278 (44.12)	1.186	0.276
ST-T changes, n(%)	949 (64.56)	402 (63.81)	0.108	0.743
CAC, n(%)	651 (44.29)	296 (46.98)	1.297	0.255
Typical chest pain, n(%)	735 (50.0)	334 (53.02)	1.605	0.205

*DM* Diabetes mellitus, *SBP*, Systolic Blood Pressure, *DBP*, Diastolic Blood Pressure, *AST* Aspartate aminotransferase, *BUN* blood urea nitrogen, *Scr* Serum creatinine, *ABG* Admission blood glucose, *UA* Uric acid, *FIB* Fibrinogen, *HGB* Hemoglobin, *LY* Lymphocyte, *MONO* Monocyte, *NE* Neutrophil, *PLT* Platelet, *RBC* Red blood cell, *WBC* White blood cell, *CAC* Coronary artery calcification

Comparative analysis of general information indicated that CAD patients were older, had a higher number of male and smoking patients, more typical chest discomfort symptoms, and a significantly higher proportion of patients with coronary artery calcification and ECG changes. Additionally, more patients had hypertension, cerebral infarction, and diabetes, and higher levels of serum aspartate aminotransferase, blood urea nitrogen, serum creatinine, and neutrophils compared to the control group (*P* < 0.05), as shown in Table 2.

**Table 2:** Comparison of clinical and biochemical data between Normal group and CAD

Characteristic	Normal group ( N=589)	CAD group ( N=881)	*Z/T/X*	*P*
Age, (years)	56 (50,65)	65 (55,72)	−10.482	<0.001
Weight, (Kg)	64 (57,73)	64 (57,71)	−0.816	0.414
Heart rate, (Bpm)	77 (68,86)	76 (68,87)	−0.096	0.923
SBP, (mmHg)	135 (125,148.5)	140 (126,154)	−3.611	<0.001
DBP, (mmHg)	85 (78,94)	84 (77,92)	−2.04	0.041
AST, (U/L)	20.6 (16.55,24.9)	20.6 (17.2,27.3)	−1.788	0.074
BUN, (mmol/L)	5.11 (4.245,6.1)	5.5 (4.57,6.77)	−5.616	<0.001
Ca, (mmol/L)	2.33 (2.26,2.4)	2.31 (2.23,2.39)	−2.686	0.007
Scr, (umol/L)	63.2(53,73.6)	69 (59.6,81.65)	−6.861	<0.001
ABG, (mmol/L)	5.29 (4.895,5.66)	5.3 (4.93,6.195)	−3.47	0.001
UA, (umol/L)	323 (266.5,376)	339 (286,401.5)	−4.275	<0.001
FIB, (mmol/L)	3.16 (2.715,3.585)	3.18 (2.81,3.8)	−2.891	0.004
HGB, (g/L)	135 (126,146)	134 (123,145)	−1.994	0.046
LY, (×109/L)	1.58 (1.265,2.01)	1.52(1.2,1.91)	−2.325	0.02
MONO, (×109/L)	0.39 (0.3,0.49)	0.41 (0.32,0.52)	−3.679	<0.001
NE, (×109/L)	3.61 (2.815,4.67)	3.87 (3.06,4.87)	−3.197	0.001
PLT, (×109/L)	196 (159,241.5)	187 (151,226)	−3.332	0.001
RBC, (×109/L)	4.53 (4.19,4.9)	4.43 (4.1,4.845)	−3.214	0.001
WBC, (×109/L)	5.89 (4.92,7.1)	6.09 (4.98,7.26)	−1.992	0.046
Male, n (%)	289 (49.07)	519 (58.91)	13.82	<0.001
Hypertension, n (%)	282 (47.88)	549 (62.32)	29.944	<0.0010
Diabetes, n (%)	57 (9.68)	190 (21.57)	35.692	<0.001
Smoking, n (%)	92 (15.62)	191 (21.68)	8.34	0.004
Cerebral Infarction, n(%)	60 (10.19)	114 (12.94)	2.564	0.109
Hyperlipidemia, n(%)	248 (42.11)	363 (41.20)	0.118	0.731
ST-T changes, n(%)	251 (42.61)	698 (79.23)	206.818	<0.001
CAC, n(%)	87 (14.77)	564 (64.02)	346.984	<0.001
Typical chest pain, n(%)	129 (21.9)	606 (68.79)	310.372	<0.001

*DM* Diabetes mellitus, *SBP* Systolic Blood Pressure, *DBP* Diastolic Blood Pressure, *AST* Aspartate aminotransferase, *BUN* blood urea nitrogen, *Scr* Serum creatinine, *ABG* Admission blood glucose, *UA* Uric acid, *FIB* Fibrinogen, *HGB* Hemoglobin, *LY* Lymphocyte, *MONO* Monocyte, *NE* Neutrophil, *PLT* Platelet, *RBC* Red blood cell, *WBC* White blood cell, *CAC* Coronary artery calcification.

### Coronary heart disease risk factor screening

Factors showing statistically significant differences (*P* < 0.2) between CAD and control groups were included in the univariate logistic regression analysis to screen for factors associated with CAD. The results indicated that age, systolic blood pressure, aspartate aminotransferase, BUN, serum creatinine, glucose, uric acid, fibrinogen, lymphocytes, monocytes, neutrophils, platelets, WBC, gender, hypertension, diabetes, smoking, ECG, ST-T changes, coronary artery calcification, and typical angina were factors associated with CAD (*P* < 0.05), as shown in Table 3.

**Table 3:** Univariate and Multivariate logistic regression analysis of influencing factors of CAD

	*B*	*Wald*	*OR(95%CI)*	*P*	*B*	*Wald*	*OR(95%CI)*	*P*
Age,	0.046	104.926	1.047(1.038–1.056)	＜0.001	0.017	6.978	1.017(1.004–1.03)	0.008
SBP	0.007	9.663	1.007(1.003–1.012)	0.002				
DBP	0.007	3.231	0.993(0.986–1.001)	0.072				
AST	0.007	7.334	1.007(1.002–1.012)	0.007				
BUN	0.156	29.978	1.169(1.106–1.236)	＜0.001				
Ca	−0.843	5.799	0.43(0.217–0.855)	0.016				
Scr	0.014	32.303	1.014(1.009–1.019)	＜0.001				
ABG	0.092	10.714	1.096(1.038–1.159)	0.001				
UA	0.002	22.388	1.002(1.001–1.003)	＜0.001				
FIB,	0.222	13.479	1.249(1.109–1.406)	＜0.001				
LY	−0.177	5.461	0.838(0.723–0.972)	0.019				
MONO	1.023	13.45	2.781(1.61–4.804)	＜0.001				
NE,	0.095	11.678	1.099(1.041–1.161)	0.001	0.098	6.513	1.103(1.023–1.189)	0.011
PLT	−0.003	14.906	0.997(0.995–0.998)	＜0.001				
RBC	−0.139	2.962	0.87(0.743–1.019)	0.085				
WBC	0.061	6.625	1.063(1.015–1.114)	0.01				
Male	0.476	26.72	1.61(1.344–1.929)	＜0.001	0.645	23.865	1.905(1.471–2.468)	0
Hypertension,	0.593	41.214	1.81(1.51–2.169)	＜0.001				
Diabetes	0.902	41.084	2.466(1.871–3.249)	＜0.001	0.64	11.357	1.896(1.307–2.75)	0.001
Smoking	0.325	7.59	1.384(1.098–1.744)	0.006				
Cerebral Infarction	0.229	2.493	1.258(0.946–1.672)	0.114				
ST-T changes	1.344	191.626	3.834(3.169–4.637)	＜0.001	1.468	119.558	4.34(3.336–5.646)	＜0.001
CAC,	2.021	321.453	7.543(6.048–9.408)	＜0.001	2.124	202.996	8.365(6.246–11.204)	＜0.001
Typical chest pain	2.417	446.126	11.21(8.958–14.028)	＜0.001	2.807	392.02	16.553(12.537–21.853)	＜0.001

*DM* Diabetes mellitus, *SBP* Systolic Blood Pressure, *DBP* Diastolic Blood Pressure, *AST* Aspartate aminotransferase, *BUN* blood urea nitrogen, *Scr* Serum creatinine, *ABG* Admission blood glucose, *UA* Uric acid, *FIB* Fibrinogen, *HGB* Hemoglobin, *LY* Lymphocyte, *MONO* Monocyte, *NE* Neutrophil, *PLT* Platelet, *RBC* Red blood cell, *WBC* White blood cell, *CAC* Coronary artery calcification

Factors with statistically significant differences (*P* < 0.2) in the univariate logistic regression results were included in the multivariate logistic regression analysis (collinearity diagnostics showed all indicators had a variance inflation factor < 10 and tolerance > 0.1) to identify independent factors associated with CAD. The results showed that age [*OR* = 1.026, 95% *CI* (1.013–1.039), *P* < 0.001], neutrophils [*OR* = 1.101, 95% CI (1.018–1.190), *P* = 0.016], male gender [*OR* = 1.786, 95% *CI* (1.372–2.325), *P* < 0.001], diabetes [*OR* = 2.227, 95% *CI* (1.557–3.331), *P* < 0.001], ST-T changes [*OR* = 8.004, 95% *CI* (6.045–10.598), *P* < 0.001], coronary artery calcification [*OR* = 11.591, 95% *CI* (8.615–15.595), *P* < 0.001], and typical chest pain [*OR* = 14.103, 95% *CI* (10.657–18.664), *P* < 0.001] were independent factors associated with CAD, as shown in Table 3.

### Construction of the CAD risk prediction model

Based on the results of the multiple logistic regression analysis, a risk prediction model for CAD was developed, defining age and neutrophil counts into segmented categories (Table 4).

**Table 4:** Parameter assignment table

Age,(years)	Assignment	NE, (×10^9^/L)	Assignment
＜30	0	＜1.8	0
30–39	1	1.80–3.79	1
40–49	2	3.80–5.79	2
50–59	3	5.80–7.79	3
60–69	4	7.80–9.79	4
70–79	5	≥9.80	5
≥80	6		

**Fig. 1:**
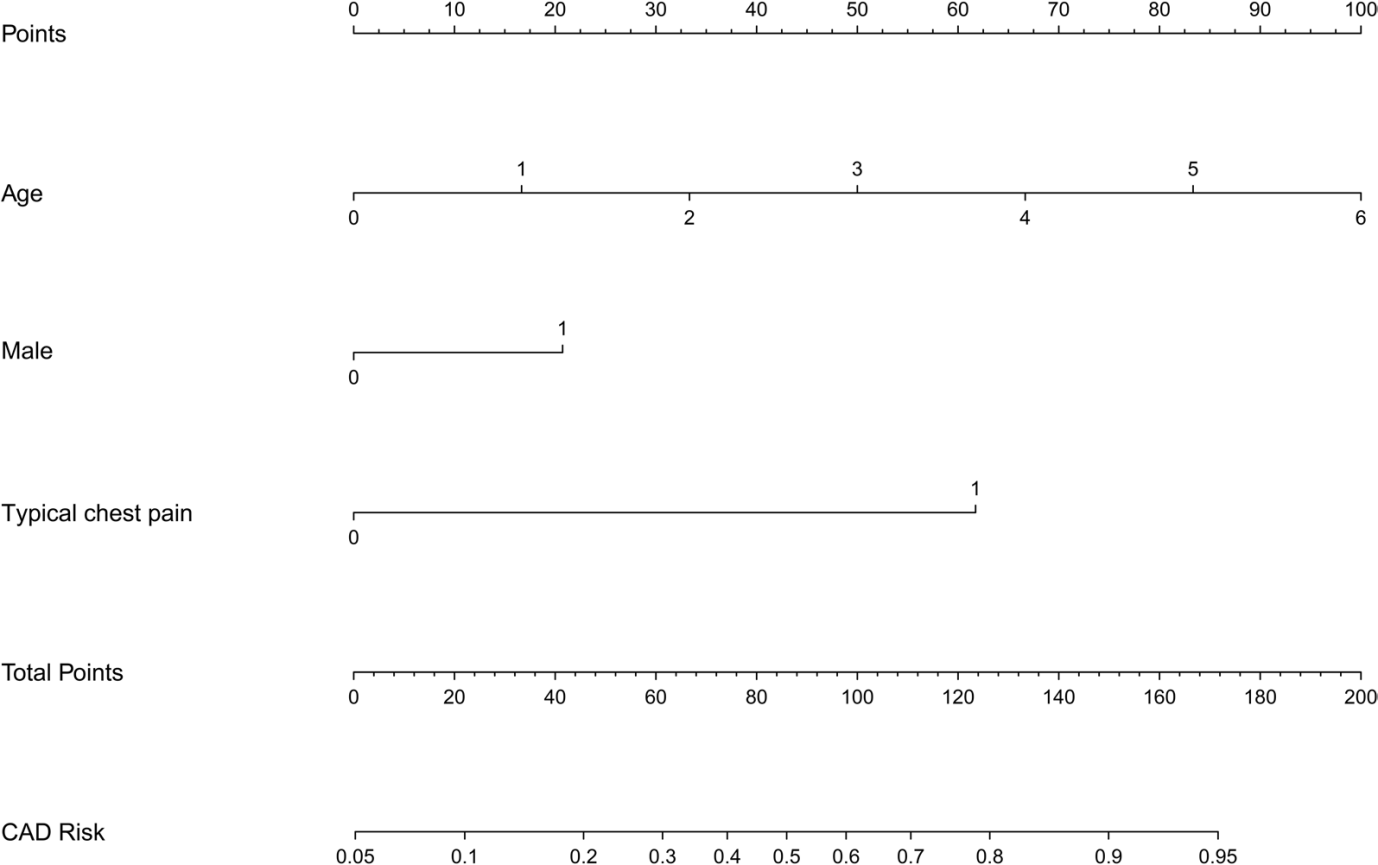
Nomogram analysis of diamond and forrester model

Model 1 (Diamond and Forrester Model): based on age, gender, and typical chest pain. The C-index of the prediction model was 0.806 (95% CI 0.783–0.828). Internal bootstrap validation with repeated sampling (1,000 repetitions) showed a C-index of 0.805. Validation in the validation cohort resulted in a C-index of 0.807 (Fig. 1).

**Fig. 2:**
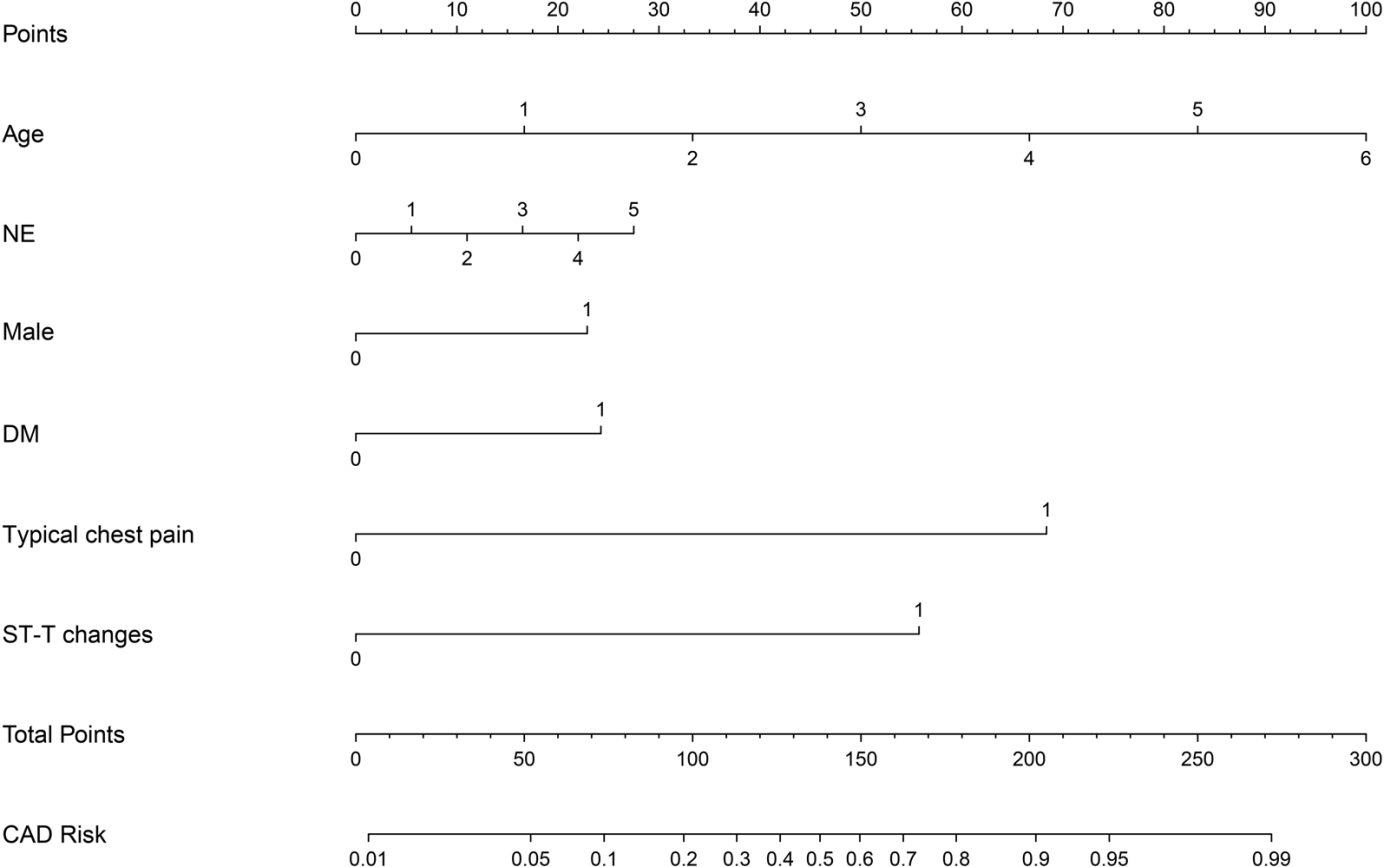
Nomogram analysis of CAD model (Primary Care Version)

Model 2 (CAD Model—Primary Care Version): Considering the clinical availability of model parameters, this model was based on age, gender, diabetes, typical chest pain, ST-T changes, and neutrophils (NE). The C-index of the prediction model was 0.866 (95% *CI* 0.841–0.880). Internal bootstrap validation showed a C-index of 0.865, and external validation resulted in a C-index of 0.861 (Fig. 2).

**Fig. 3:**
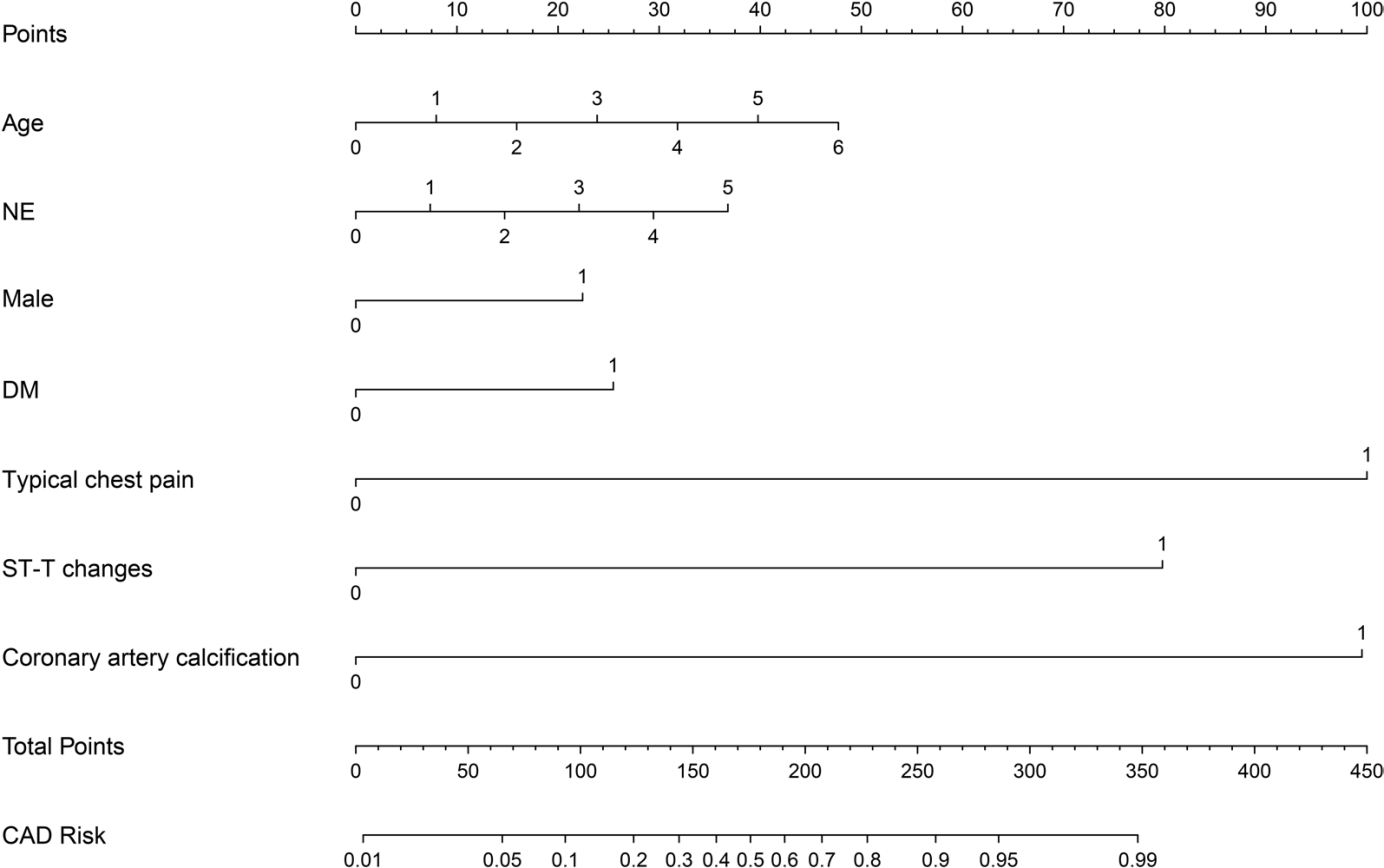
Nomogram analysis of CAD model

Model 3 (CAD Model): based on age, diabetes, neutrophils (NE), male gender, ST-T changes, coronary artery calcification, and typical chest pain. The C-index of the prediction model was 0.909 (95% CI 0.894–0.925). Internal bootstrap validation showed a C-index of 0.907, and validation in the validation cohort resulted in a C-index of 0.897 (Fig. 3).

**Fig. 4:**
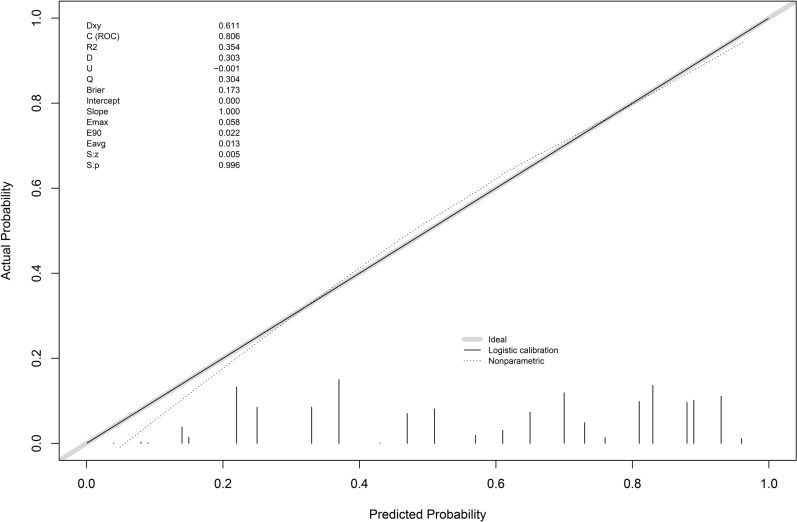
Diamond and forrester model calibration curve

**Fig. 5:**
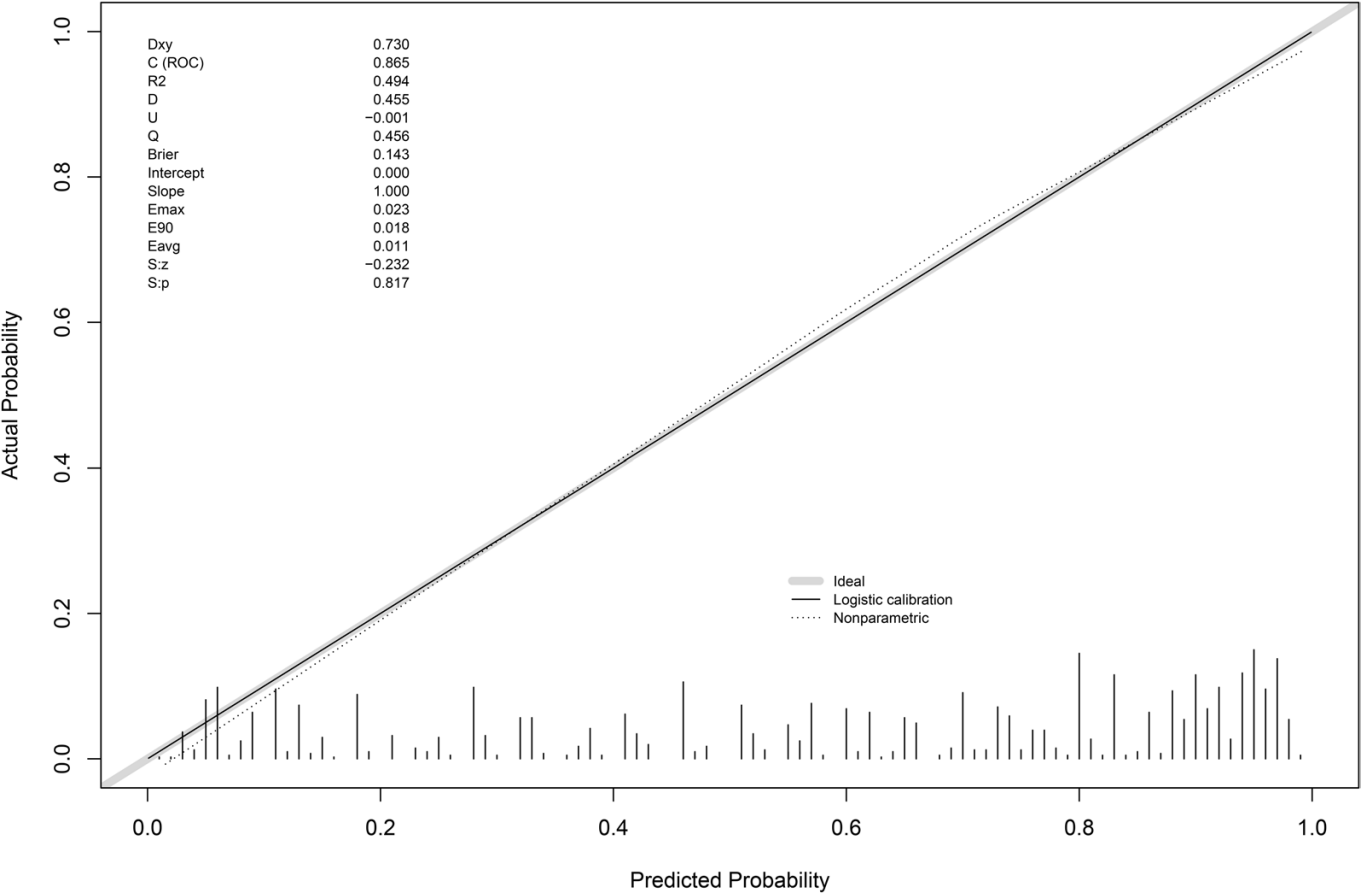
CAD model (Primary Care Version) model calibration curve

**Fig. 6:**
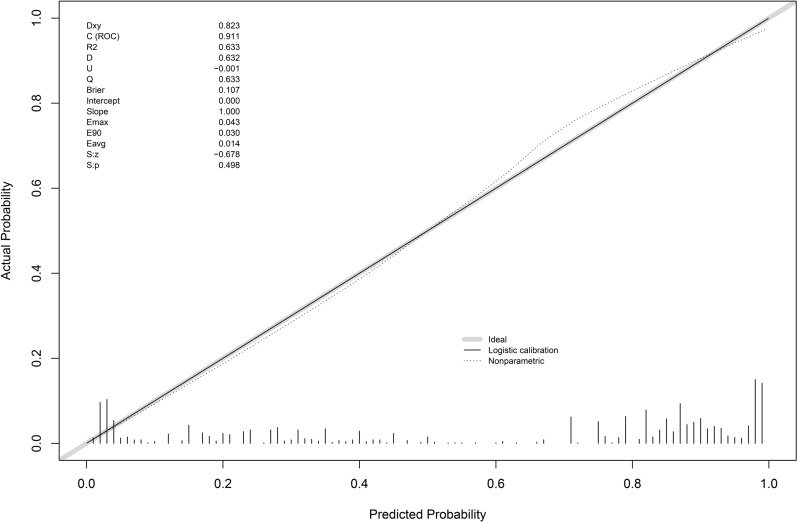
CAD model calibration curve

Calibration and Validation: calibration curve analysis indicated that the nomogram models had good calibration, with slight fluctuations around the ideal model curve (diagonal line) (Figs. 4, 5, 6). External validation also showed good calibration (Figures S1, S2, S3). Based on the results of models 2 and 3, clinical scoring tables were constructed (Tables 5, 6).

**Table 5:** Coronary artery disease risk assessment tool (primary care version)

Age	Score	NE, (×10^9^/L)	Score	Characteristic	Score
＜30	0	＜1.8	0	Male	23
30–39	17	1.80–3.79	6	DM	24
40–49	33	3.80–5.79	11	ST-T changes	56
50–59	50	5.80–7.79	17	Typical chest pain	68
60–69	67	7.80–9.79	22		
70–79	83	≥9.80	28		
≥80	100				

*NE* Neutrophil, *DM* Diabetes mellitus, *CAC* Coronary artery calcification

**Table 6:** Coronary artery disease risk assessment tool

Age	Score	NE, (×10^9^/L)	Score	Characteristic	Score
＜30	0	＜1.8	0	Male	22
30–39	8	1.80-3.79	7	DM	25
40–49	16	3.80-5.79	15	ST-T changes	80
50–59	34	5.80-7.79	22	CAC	99
60–69	32	7.80-9.79	29	Typical chest pain	100
70–79	40	≥ 9.80	37		
≥80	48				

*NE* Neutrophil, *DM* Diabetes mellitus, *CAC* Coronary artery calcification

ROC curve analysis was used to evaluate the predictive value of each model for CAD.

Model 1: At a cut-off score of 97.10, the model had the highest predictive efficiency for CAD, with a corresponding CAD risk of 60%, sensitivity of 74.3%, specificity of 73.3%, and AUC of 0.806 (95% *CI* 0.783–0.828, *P* < 0.01).

Model 2: At a cut-off score of 149.99, the model had the highest predictive efficiency for CAD, with a corresponding CAD risk of 60%, sensitivity of 79.8%, specificity of 77.9%, and AUC of 0.865 (95% *CI* 0.846–0.884, *P* < 0.01). Based on the ESC 2019 guidelines, if CAD risk is greater than 60%, coronary angiography or CTA is recommended; if CAD risk is between 5 and 60%, observation in the emergency department is recommended; if CAD risk is less than 5%, follow-up observation is suggested.

Model 3: At a cut-off score of 185.38, the model had the highest predictive efficiency for CAD, with a corresponding CAD risk of 56%, sensitivity of 87.4%, specificity of 84.9%, and AUC of 0.911 (95% *CI* 0.896–0.927, *P* < 0.01). If CAD risk is greater than 57%, hospitalization for coronary angiography or CTA is recommended; if CAD risk is between 5 and 57%, observation in the emergency department is recommended; if CAD risk is less than 5%, follow-up observation is suggested.

### Model performance evaluation

**Fig. 7:**
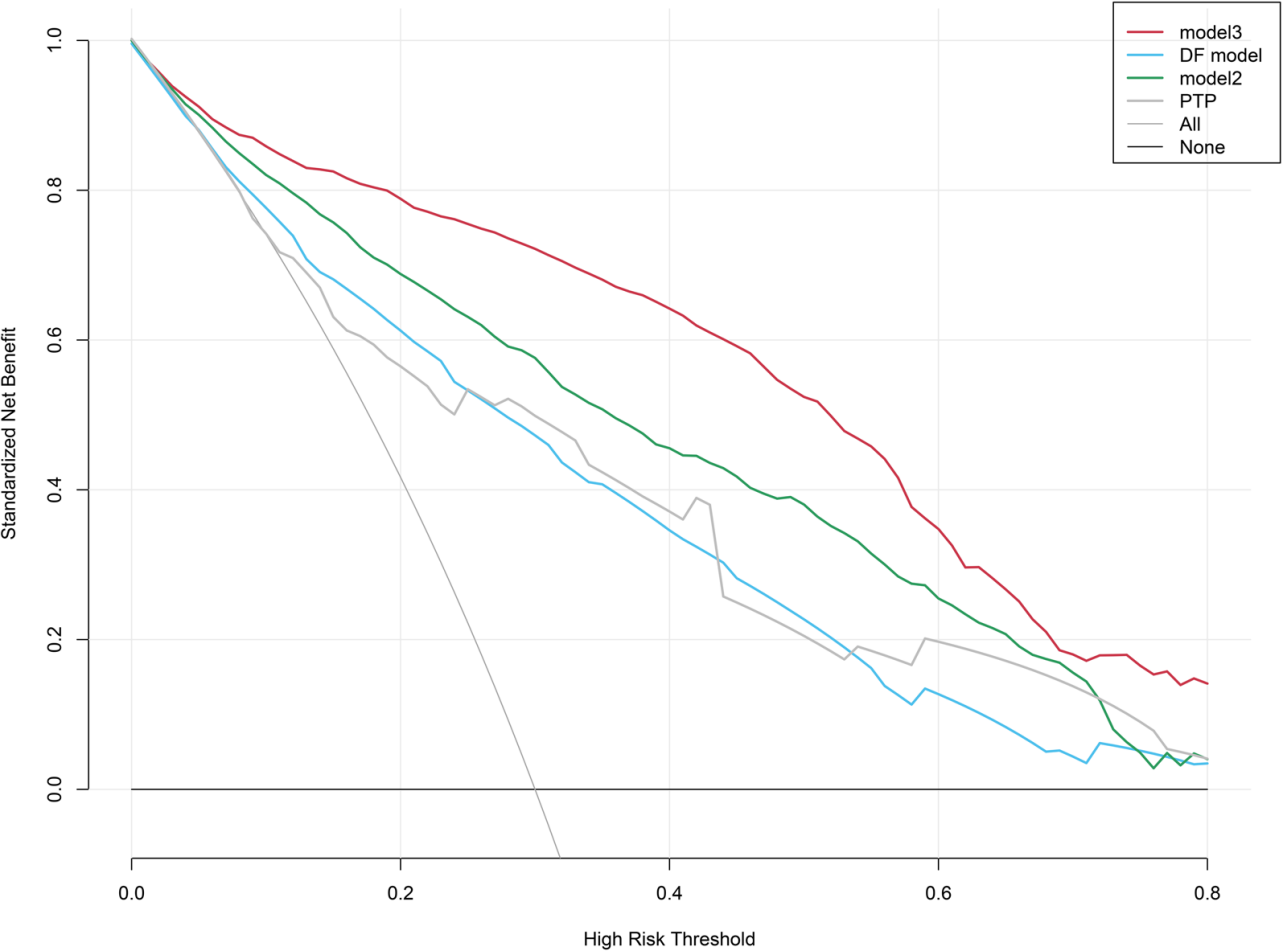
DCA curve analysis of the prediction model for CAD

Using the 2019 guideline-recommended PTP calculation method, we calculated PTP for all patients and compared the clinical performance of Model 1 (Diamond and Forrester Model), Model 2, Model 3, and the PTP model using DCA curves. In the DCA curves, Pt represents the threshold probability of obstructive CAD in the local population. When Pt is between 0 and 0.80, Model 3 showed higher clinical net benefit compared to Model 2, the PTP model, and the DF model, indicating significant advantages in clinical practice (Fig. 7). External validation results also showed that Model 3 had significantly higher clinical net benefit compared to Model 2, the PTP model, and the DF model (Figure S4). Additionally, we found that the PTP model outperformed the DF model in a larger Pt interval, consistent with previous studies. Although Model 2's clinical net benefit was lower than Model 3, it was still significantly higher than the DF and PTP models under relatively limited conditions, indicating its usability in primary hospitals.

## Discussion

Cardiovascular diseases are the leading cause of death globally, with approximately 17.9 million deaths annually, accounting for 32% of all global deaths, of which about 45% [19] are due to coronary atherosclerotic heart disease. In clinical practice, many patients with CAD initially present with symptoms such as chest pain and visit various hospital departments. Among these patients, up to 15% with chest pain have CAD, including angina and myocardial infarction. This proportion increases to 22% in emergency departments and 28% [20–22] in cardiology clinics. However, due to various reasons, these patients may not receive adequate attention and timely intervention, leading to disease progression and serious consequences [4]. Chest pain is one of the most common symptoms of coronary artery disease, but it can also be caused by many other factors such as gastrointestinal diseases, musculoskeletal problems, and psychological factors [23]. Therefore, effectively identifying and assessing the risk of chest pain patients in an outpatient setting is a significant challenge for clinicians.

Therefore, by collecting available data from outpatient and emergency departments, we used logistic regression analysis to screen for factors related to coronary artery disease and constructed a CAD prediction model. Unlike previous CAD prediction models, our model introduces inflammation parameters for the first time. Inflammation is an important risk factor for CAD, and its role in the development of CAD has received increasing attention in recent years [24]. However, previous CAD risk prediction models often overlooked this parameter. In our study, we found that NE is an independent factor related to CAD, and we constructed a risk prediction model based on NE and other parameters. The introduction of inflammation parameters allows our model to more comprehensively assess the risk of CAD, providing a more accurate risk prediction tool.

The Diamond and Forrester model is a guideline-recommended CAD risk prediction model based primarily on age, gender, and symptoms [25]. Numerous studies have shown that it tends to overestimate the probability of CAD occurrence and does not consider cardiovascular risk factors associated with the disease. Although Tessa S S Genders and colleagues improved the model by adding CAD-related risk factors and coronary artery calcification scores, greatly increasing its predictive accuracy, the final model's predictive probabilities need to be calculated online [26]. As Tessa S S Genders et al. noted, this risk prediction model can be networked in electronic medical records, electronic prescription entry systems, or smartphone or tablet applications, which is not applicable to the majority of large outpatient and emergency departments in China and is even less feasible for smaller community hospitals.

Our study constructed a CAD risk score based on neutrophils, CT coronary calcification, electrocardiogram (ECG), and typical chest pain parameters. Compared to the Diamond and Forrester model, the parameters required for the score, such as neutrophils, CT coronary calcification, and ECG, are usually quickly obtainable. Additionally, unlike the improved model by Tessa S S Genders et al., where coronary artery calcification scores often require estimation by specialized imaging physicians, the determination of CT coronary calcification is relatively simple. Clinicians can obtain the results by reading the images before the report is issued, allowing for rapid assessment of CAD risk. This model's application is not limited to large hospitals; it also has promotional value in resource-limited primary healthcare institutions. The inflammation parameters can be easily obtained through simple blood tests, making the model's application more convenient and widespread.

Additionally, most of the current CAD risk assessment models are developed based on Western populations, and their applicability to the Chinese population is unknown. Therefore, we constructed a CAD risk prediction model suitable for the Chinese population based on data from two cohort studies and tested it against the Diamond and Forrester model. We found that, similar to the 2019 guideline-recommended model, the Diamond and Forrester model also overestimated the risk of CAD, indicating the necessity of developing a CAD risk prediction model suitable for the Chinese population. The Diamond and Forrester model was developed over 40 years ago for patients aged 30–70 years. With the advancement of healthcare, the average life expectancy in China has significantly increased. For patients over 70 years old, the Diamond and Forrester model has significant limitations. Our study included patients over 70 years old, accounting for 25.42% of the population, and the model is also applicable to this older age group.

### Research innovation

We introduced inflammation parameters, particularly NE, into the CAD risk prediction model for the first time, enhancing the model's predictive ability for CAD and making the assessment more comprehensive and accurate. Our model was developed and validated based primarily on the Chinese population, addressing the shortcomings of existing models in the Chinese population. DCA curve analysis demonstrated the practicality and effectiveness of the new model. We constructed two clinically applicable risk score tables using the nomogram model, with easily obtainable parameters, making the model suitable for both large hospitals and resource-limited primary healthcare institutions. Compared to the Diamond and Forrester model, our model extends its applicability to include patients over 70 years old, addressing the limitations of existing models. We conducted internal and external validations of the prediction model, accurately assessing its stability and reliability to ensure its practicality across different datasets and clinical environments.

### Limitations of the study

This study has the following limitations: 1. Our model was developed and validated primarily based on a single-center Chinese population. Although our model addresses the shortcomings of existing models in the Chinese population, its generalizability to other populations has not been verified, which may limit its widespread application. 2. Our data are sourced from a single center, which may introduce selection bias. 3. Although our study conducted external validation, the data source is singular, and the sample size is still not large enough. Further expansion of the sample size and multi-center studies are needed. These limitations indicate that we still need to continuously optimize the model parameters in future research and practice.

## Conclusion

In summary, our study successfully developed a new CAD risk prediction model. Compared to previous CAD risk prediction models, our model introduced inflammation parameters, improving the accuracy and comprehensiveness of CAD risk assessment. In addition, the novel model was mainly based on the data of the Chinese population, which solves the current situation of insufficient data of Chinese patients in the previous prediction model. DCA curve analysis demonstrated that the clinical net benefit of the new model is superior to that of other traditional models.

## Supplementary Information

Supplementary material 1

Supplementary material 2

Supplementary material 3

Supplementary material 4

## Data Availability

The datasets used and/or analyzed in this study are available from the corresponding author upon reasonable request.

## Abbreviations

ABG: Admission blood glucose
ANOVA: Analysis of Variance
AST: Aspartate aminotransferase
BUN: Blood urea nitrogen
CAC: Coronary artery calcification
CAD: Coronary artery disease
CAG: Coronary angiography
CI: Confidence interval
CPU: Chest pain units
CT: Computed tomography
DBP: Diastolic blood pressure
DCA: Decision curve analysis
DM: Diabetes mellitus
ECG: Electrocardiogram
FIB: Fibrinogen
HGB: Hemoglobin
LY: Lymphocyte
MONO: Monocyte
NE: Neutrophils
OR: Odds ratio
PLT: Platelet
PTP: Pre-test probability
RBC: Red blood cell
SBP: Systolic blood pressure
Scr: Serum creatinine
SD: Standard deviation
UA: Uric acid
WBC: White blood cell
